# The ethics of Wegovy: promoting autonomy in pediatric care

**DOI:** 10.1007/s11019-025-10300-8

**Published:** 2025-10-09

**Authors:** Nanette Ryan, Julian Savulescu

**Affiliations:** 1 Centre for Biomedical Ethics, Yong Loo Lin School of Medicine, National University of Singapore, Singapore, Singapore; 2 Uehiro Oxford Institute, University of Oxford, Oxford, UK

**Keywords:** Autonomy, Wegovy, Ozempic, Weight-loss, Ethics, Children, Paediatrics, Obesity

## Abstract

Semaglutide, marketed as Wegovy, Ozempic, and Rybelsus, is a glucagon-like peptide-1 receptor agonist (GLP-1 RA) that has attracted significant global attention for its appetite-suppressing and weight-loss effects. Approved for pediatric use in children aged 12 and older, Wegovy has been described as a “miracle drug” and hailed as a potential solution to the so-called “obesity epidemic.” However, prescribing medication to children raises complex ethical questions, including how best to respect young patients’ autonomy and promote their well-being. This paper focuses on one such concern: the “argument from virtue.” According to this view, Wegovy represents a morally problematic approach to weight loss because it circumvents the development of character traits—such as self-control and resilience—that are seen as integral to autonomy. We critically examine this argument, rejecting the claim that there is a single morally “right way” to lose weight, and argue that Wegovy, when prescribed within a supportive framework, may help children build and exercise autonomy. We review the drug’s function and efficacy, analyze how it might promote or hinder autonomy, and offer recommendations to support ethical prescribing practices that align with respect for children’s agency.

## Introduction

Semaglutide, marketed as Wegovy, Ozempic, and Rybelsus, is one of the newest glucagon-like peptide-1 receptor agonists (GLP-1 RAs) developed by Novo Nordisk. Originally developed for type 2 diabetes, it has gained global attention for appetite suppression and weight loss, with some heralding it as a “miracle drug,” and predicting it will end the “obesity epidemic.” Wegovy was first approved in 2022 by the U.S. Food and Drug Administration (FDA) for children aged 12 and older to treat pediatric obesity, following the success of the STEP TEEN trial (Weghuber et al. 2022). Since then, many countries, including Germany, the U.K., Denmark, and the U.A.E., have also approved Wegovy for pediatric weight loss (Respuat and Terhune 2024).

Despite the medication’s wide regulatory approval, responses to its use in pediatric care have been mixed. Some commentators argue that caution is needed when prescribing Wegovy to children, given the lack of data on its effects on children’s development or its long-term safety and efficacy. Dr. Dan Cooper, professor of pediatrics at UC Irvine, notes, “We don’t really know what these medications do in the context of the growing child (Respuat and Terhune 2024). Tamara Hannon, a pediatric endocrinologist at Indiana University, adds, “Although adults face many of the same unknowns, the risks for teens could be more severe because of their physiological pubertal development” (Tayag 2023). Others argue that Wegovy is an underutilized treatment for childhood obesity. Dr. Suzanne Cuda, director of Alamo City Healthy Kids and Families, says, “I use these medications wherever I can. Unlike with adults, where it’s more of a rescue operation, we are much more likely with children and adolescents to prevent disease” (Respuat and Terhune 2024).

In addition to these concerns, many are worried about the wider social implications of Wegovy use on pediatric and adult health. Rosenfield (2024), author of “Why Ozempic Is Cheating”, argues that even if weight loss with Wegovy achieves positive health outcomes for patients, they are “ill-gained” rewards. Rosenfield states (2024), “Whatever one’s reasons for losing weight, the common wisdom is that it’s not supposed to be easy, physically or mentally”. According to this view, weight loss should be achieved the “right way,” through diet and exercise. Fitness trainer and American media personality Jillian Michaels has also spoken out against the use of Ozempic and medicalized weight loss interventions, calling them “the easy way out” (Gordon and Hobbes 2025). Michaels claims that Ozempic “is being wrongly utilized as a shortcut for weight loss” (Herz 2025). These sentiments are echoed by some parents and children seeking obesity treatment. In their exploratory study on the attitudes and normative beliefs of specialists, parents, and adolescents with obesity, van Geelen et al. (2013: 227) found that in some cases both parents and adolescents described medicalized weight-loss interventions as the “wrong way” to lose weight, emphasizing a need to “control their own condition” without medicalized intervention. One teen reflecting on bariatric surgery stated, “Nine out of ten times it’s your own fault… I’m convinced that when I decide for myself that it is the time to truly lose weight, I will be able to do it. And in the end, I will be much happier that I was able to do it without the help of surgery” (van Geelen et al. 2013: 233).

Prescribing medication to children ethically calls for careful analysis of several important considerations, including the medication’s safety and efficacy for the pediatric community; how its use respects the individual patient’s needs and choices; and how its prescription aligns with broader public health goals. In this paper, our focus is not on the potential medical harms to children from taking Wegovy, but on what we will refer to as the “argument from virtue”. The argument from virtue holds that there is a correct—or “virtuous”—way to lose weight, as Rosenfield describes, and that Wegovy is problematic not only because it represents the “wrong way” to lose weight, but also because it interferes with the development and expression of virtuous character traits. Such traits are arguably valuable in themselves and integral to the exercise of autonomy. As respect for autonomy is widely regarded as a pillar of ethical healthcare, if the argument from virtue is correct, it would provide a strong *prima facie* reason against prescribing Wegovy—particularly in the case of children, to whom special duties of moral and agency-related support are owed.

In what follows, we reject the claim that there is a morally “right way” to lose weight but concede that Wegovy could be prescribed in ways that harm the development and exercise of children’s autonomy. However, when provided within a supportive and respectful environment, we argue that Wegovy has the potential to be an important tool to help children develop and express their autonomy.

We begin by outlining the function and efficacy of Wegovy for weight loss and health outcomes in children. We assess the argument from virtue and describe how Wegovy may be used to promote children’s autonomy. We conclude by identifying potential barriers to success and offering recommendations to better support autonomy in pediatric care.

This paper focuses on Wegovy (semaglutide), currently the most widely recognized GLP-1 receptor agonist on the market. Other GLP-1 RAs approved for weight management include Saxenda (liraglutide) and Zepbound (tirzepatide). To date, only Wegovy and Saxenda have been approved for use in pediatric populations. Given that these medications share similar mechanisms of action and clinical outcomes, the arguments advanced here extend beyond Wegovy to encompass GLP-1 RAs more generally, and may also be relevant in some ways to the use of bariatric surgery in pediatric care.

We focus on children aged 12 years and older, the approved age group for Wegovy in countries where it is authorized for pediatric use. We use the terms “youth” and “adolescent” to refer to those aged 12 to 18.

## The science + potential

According to Novo Nordisk, Wegovy is for “adults and children aged 12 and older with obesity, or some adults with overweight who also have weight-related medical problems, to help them lose excess body weight and keep the weight off” (Novo Nordisk 2024a). Youth aged 12 and older take Wegovy as a weekly single-dose injection, starting at 0.25 mg for the first four weeks and increasing to a 2.4 mg maintenance dose.

Wegovy promotes weight loss by suppressing appetite. It slows gastric emptying and interacts with brain signaling related to hunger and satiation, effectively silencing experiences of hunger as well as rumination and preoccupation with food—so-called “food noise” (Hayashi et al. 2023). As we have described elsewhere, how Wegovy interacts with the brain and brain chemistry remains uncertain (Ryan and Savulescu 2025). There is speculation that because GLP-1 RAs remain in the bloodstream, they may cross the blood-brain barrier to access deeper parts of the brain—an idea that has prompted excitement about their potential to treat conditions such as neurodegenerative disorders, multiple sclerosis, and endotoxemia (Tipa et al. 2024; Zhang 2024). Evidence for this, however, is limited and speculative. Some studies suggest semaglutide affects the brainstem, septal nucleus, and hypothalamus, interacting through circumventricular organs and sites near the ventricles that monitor and respond to blood composition (Gabery et al. 2020). The role and significance of this interaction remain unclear.^1^

Results in adolescents taking Wegovy for weight loss have been substantial. In the 68-week STEP TEENS randomized controlled trial of 180 participants aged 12 to under 18 years with obesity or overweight and at least one weight-related condition, those receiving Wegovy along with diet and exercise lost an average of 16.1% of body weight, compared to 0.6% in the placebo group (Weghuber et al. 2022). 44% of participants on Wegovy were reclassified to a normal or overweight BMI, versus 12.1% in the placebo group (Kelly et al. 2023). In addition to weight reduction, the STEP TEENS trial reported improvements in health indicators linked to future cardiovascular risks and childhood non-alcoholic fatty liver disease (NAFLD) (Weghuber et al. 2022: 2255).^2^

Common side effects include nausea, vomiting, diarrhea, abdominal pain, bloating, constipation, headaches, loss of lean muscle mass, and exercise-related weakness. In the STEP TEENS trial, 62% of participants in the semaglutide group experienced mild or moderate nausea, vomiting, and diarrhea, compared to 42% in the placebo group (Weghuber et al. 2022: 2245). Serious side effects were reported in 11% of the semaglutide group and 9% of the placebo group. Adverse events led to trial discontinuation in 5% of the semaglutide group and 4% of the placebo group.

More serious, though rare, side effects have been documented in adult trials, including gastroparesis (stomach paralysis) and anhedonia (depression). Potential long-term risks include thyroid C-cell tumors (adenomas and carcinomas), pancreatitis, kidney failure, gallbladder disease, and diabetic retinopathy (Suran 2023: 1628; Llamas 2024). Weight regain is also common when patients stop treatment. In the STEP 1 trial extension involving 1,961 participants with a BMI of 30 kg/m² or higher, or 27 kg/m² or higher with at least one weight-related comorbidity and no diabetes, Wilding et al. (2021)found that participants receiving once-weekly subcutaneous semaglutide (2.4 mg) and lifestyle intervention regained two-thirds of their prior weight loss within one year of stopping treatment, along with similar reversals in cardiometabolic variables.

Long-term side effects and potential risks for children have not yet been observed in clinical trials. However, the same risks identified in adults—including weight regain—are listed as product warnings for adolescent use of Wegovy (Novo Nordisk 2024b). Malnutrition is also listed as a potential risk for both pediatric and adult populations (Miller 2024).^3^

## Children’s autonomy in theory

Beauchamp and Childress’ (2019) conception of autonomy is a central pillar of biomedical ethics. It is grounded in the Western philosophical tradition, where self-governance is central. In their view, personal autonomy requires liberty—or self-rule that is free from controlling influences by others and from limitations, such as understanding—and agency—or the “capacity for intentional action” (2019: 100).

On many philosophical accounts of autonomy, such as those in the Kantian tradition, self-governance requires that one have an “established practical identity” (Korsgaard 2009: 22–24). An established practical identity is a conception of the self that is coherent, sustained over time, and reflects what we consider to be ineliminable aspects of ourselves, or our “identity-conferring commitments” (Williams 1981). It is also the framework from which a person issues authoritative autonomous decisions—it is “the self from which one governs.” Many children, as Schapiro (1999) aptly describes, do not have established practical identities or the capacities for reflection and comprehension required to issue authoritative action. For this reason, some theorists, such as (Feinberg 1980), argue that children do not yet possess autonomy. According to Feinberg, what children do have are “autonomy rights held in trust”—rights that protect their future autonomy as adults and preserve their right to an open future. A child’s future is “open” when the development of their agency-related capacities is supported and opportunities are not prematurely foreclosed, so that upon reaching maturity they are left with “as many open options, opportunities, and advantages as possible” (Feinberg 1980: 130).

Other theorists, such as Amy Mullin (2007, 2019) and Noggle (2002), hold a gradualist perspective and argue that children can have autonomy in childhood, though full autonomy is not gained until maturity. On Mullin’s (Mullin 2019: 231) view, for example, even very young children can act autonomously in ways that demonstrate “governance in accordance with personally meaningful goals and commitments.” According to Mullin, children act with self-governance when their decisions express their “volitional self.” A volitional self is “the part of the self that seeks to govern its own actions to accord with what it values, cares about, wants to accomplish” (Mullin 2007: 573). Mullin explains that children care deeply about some things—such as who they love—that constitute aspects of their volitional self. When children act with sufficient impulse control in pursuit of goals that express their volitional self, Mullin holds that they act autonomously.^4^

Robert Noggle endorses a different gradualist view, arguing that while children often lack full autonomy, they—especially during adolescence—may have capacities for agency that rival those of some autonomous adults. According to Noggle, most children lack full autonomy because they do not possess a sufficiently developed moral agency, which he sees as constitutive of full autonomy.^5^

In their *Principles of Biomedical Ethics*, Beauchamp and Childress (2019: 59) do not outline what autonomy amounts to in childhood, and they cast autonomous action as the action of “normal choosers who act (1) intentionally, (2) with understanding, and (3) without controlling influences that determine their actions” (2019: 102). Importantly, however, for the reasons outlined above, children are not “normal choosers” but are often—borrowing Robert Noggle’s (2002) term—“special agents”: persons whose capacities for autonomy vary and are still developing across different domains over time. As such, if we are to respond appropriately to the nature of a child’s autonomy and endorse a gradualist perspective, respecting that autonomy requires recognizing both children’s present capacities and their ongoing development toward future autonomy.

For the remainder of this paper, we will consider how Wegovy may promote or hinder children’s volitional autonomy—acting toward things they stably care about in local settings—as well as their future mature autonomy—acting toward their moral and identity-reflecting commitments in adulthood.

## Children’s autonomy in action

Accounts of autonomy that focus on adults typically hold that respect for a person’s autonomy places demands on others—often in the form of perfect negative duties and imperfect positive duties. Perfect duties are strict obligations that apply in most circumstances and include the requirement not to interfere with another person’s autonomy—for instance, by exerting “controlling influences” that determine their actions. Imperfect duties, by contrast, are flexible obligations that allow discretion regarding when and how they are fulfilled, and they call on individuals to support others in pursuing their autonomous ends.

Plausibly, respect for children’s autonomy gives rise to similar duties: first, to refrain from interfering with their autonomy within domains in which they are competent to choose—what Schapiro (1999) refers to as “domains of discretion”—and second, to promote both the exercise of their autonomy and the development of their capacities for agency.

In order to exercise autonomy well, persons of all ages require not only non-interference, but also real options to choose from, knowledge to make informed decisions, and, at times, the resources necessary to carry those decisions through. One way to promote—and so respect—a child’s autonomy then is to provide both knowledge and material support. Another is to offer autonomy-focused education by scaffolding or modeling good decision-making, supporting the development of character traits and capacities that underpin autonomous agency—such as impulse control, determination, and resilience—and by giving children opportunities to practice decision-making and “autonomy virtues” in supportive environments. Conversely, a child’s autonomy may be undermined by the denial of such resources and opportunities, by modeling poor decision-making, or by unjustly interfering with their choices.

## The argument from virtue

One of the key arguments against the use of Wegovy is that it is at odds with virtues of character and in particular, some of those virtues that are necessary for autonomous action.

The argument from virtue contends that despite Wegovy’s health benefits, using it for weight loss is wrong because it constitutes a form of “cheating.” Weight loss, the argument goes, is a reward for physical and mental effort—traits that express virtuous character—and those who avoid that effort receive undeserved, ill-gotten rewards. Wegovy is seen as “taking the easy way out” because it circumvents the effort and discipline needed to “deserve to be thin.” Those taking Wegovy are cast as unvirtuous and lazy cheaters who “medicate for self-control” rather than develop the virtue themselves. As critic Jillian Michaels contends, when you take Wegovy, “you’ve not learned anything. You’ve not built any physical strength or endurance. You haven’t learned to eat healthy” (Herz 2025).

The worry for autonomy is that Wegovy both discourages the expression of virtues necessary for autonomous action and encourages morally corrupt behavior at odds with moral agency—agency that Noggle contends is necessary for full autonomy.

The worry for children’s autonomy is that by prescribing Wegovy, healthcare providers and parents hinder the development and expression of children’s autonomy by facilitating their “cheating” and “laziness” and by failing to educate them in virtue and healthy living without medicalization. In doing so, they fail in their fiduciary duties to guide and educate children. Healthcare providers and parents who give Wegovy to children then are also accused of “taking the easy way out,” shirking their moral responsibilities in favor of a “quick fix” for childhood obesity.

If respect for autonomy is paramount to ethical healthcare, and the argument from virtue is correct, this would provide strong reason against prescribing Wegovy to children.

## Rejecting the argument from virtue

### Wegovy is not an easy way out

The argument from virtue is, however, unpersuasive for a number of reasons. First, as we have argued elsewhere, while taking Wegovy improves a person’s ability to lose weight, it is not an “easy way out” (Ryan and Savulescu 2025). Clinical results indicate that successful, sustained weight loss with Wegovy requires an ongoing commitment to medical intervention, as weight loss and the associated health benefits persist only as long as the medication is taken (Suran 2023). Sustained treatment requires adhering to a regimen of potentially painful and—especially for children—anxiety-inducing weekly subcutaneous injections. It is also common for adults and children to experience unpleasant side effects such as nausea and diarrhea, and to assume the risks of more serious side effects and unknowns. In addition, numerous studies highlight that weight loss is most effective when medication is combined with regular exercise and a calorie-restricted, nutritionally balanced diet—regimens that often demand fastidious planning, time-consuming monitoring, sustained energy deficits, and impulse control to execute. While children may not be directly involved in planning and monitoring—although this would be ideal for learning—they must still commit to regular physical activity and cope with calorie restriction. This requires self-control, determination, discipline, resilience, and even bravery—traits that can support children’s autonomy rather than undermine it.

### Weight loss and effort are not inherently valuable

Another reason to reject the argument from virtue is that even if taking Wegovy requires less effort to lose weight than traditional lifestyle interventions—it does, after all, improve a person’s ability to lose weight, potentially making the process easier for some, especially when traditional interventions have failed—it is by no means evident that taking Wegovy should be considered the “less virtuous” way to lose weight. This is because neither weight loss nor effort are inherently valuable or virtuous.

The value of weight loss, as with many ends, is instrumental and contingent on the needs and aims of individuals. If a person values their health and weight loss improves it, then weight loss may be valuable as a means to that end. Weight loss, however, does not necessarily lead to improved health—an assumption the argument from virtue appears to make. For some people, such as those with anorexia nervosa, weight loss not only fails to improve health but is positively harmful. For others, including those with metabolically healthy obesity (MHO), weight loss may offer no health benefits at all. Thus, weight loss is neither inherently valuable nor necessarily instrumentally valuable.

In addition, even if weight loss were necessarily tied to improvements in health, it does not follow that its instrumental value makes it the end that must be prioritized above all others. According to Thomas Hill (1991), self-respect is the crown jewel of moral character, and to act virtuously we must prioritize ends that accord with our self-respect. Part of having self-respect in maturity is cultivating a practical identity—a conception of the self that is coherent, sustained over time, and reflects what we see as ineliminable aspects of ourselves. Priority should go to ends that reflect those aspects, even if they do not include health. For children, priority should be given not to ends that necessarily contribute to their practical identities or are sustained over time, but, following Mullin, should be given to ends that reflect what they deeply care about, although duties of paternalism may justifiably interfere with those ends in favor of health.^6^  

Effort itself is not inherently virtuous or praiseworthy (Maslen et al. 2020). Excessive effort toward an end when unnecessary may be foolhardy, while efficient effort may reflect wisdom and deserve praise. The normative assessment of effort often depends less on how necessary it is to achieve an end and more on the rules governing appropriate action and the perceived value of the end itself. The effort expended on torturing someone, for example, is not considered valuable—even if torture methods like nail pulling require determination and diligence—because the end itself is morally abhorrent.

Rosenberg and others contend that weight loss must—as a rule—be achieved and deserved through hard work, diet, and exercise. The insistence on this method, however, unfairly privileges naturally thin individuals whose biological composition, all else being equal, allows them to maintain a lower body weight without equivalent effort or intervention (Loos and Yeo 2022). It also privileges those in higher socioeconomic positions, who typically have greater access to education, the resources to afford nutrient-dense foods, and the leisure time needed to diet and exercise the “correct way.” In doing so, the rule ignores well-established facts about biology and structural inequalities that contribute to the root causes of obesity and reinforces oppressive weight and class stigma that equates fatness with poor moral character. 7

Strikingly, then, insofar as the Rosenberg rule encourages stigma and ignores pertinent facts about biology and the social determinants of obesity, it appears that the rule itself may interfere with a person’s autonomy by compromising their ability to make informed, uncoerced healthcare decisions or seek treatment that might otherwise benefit them.

So, while taking Wegovy may make it easier for children to lose weight and reduce the need to develop and exercise impulse control, there are many other opportunities to build this capacity and other autonomy supporting virtues. Some of which are related to weight loss (e.g., exercise and a healthy diet that accompany Wegovy treatment), and others are unrelated (e.g., study, hobbies). Forgoing Wegovy, then, is neither necessary nor sufficient for developing virtues such as self-control or other virtues that support autonomy in childhood, and buying into the argument from virtue may be positively harmful towards those ends.

### The risk of over-medicalization

The argument from virtue also assumes that Wegovy will be prescribed to children instead of recommending and supporting lifestyle interventions, thereby depriving them of the knowledge needed to live well without medical dependence. For all the argument’s flaws, this risk is worth acknowledging: parents and healthcare providers could choose medical intervention over education and support for virtues, and in doing so, deprive children of the knowledge and capacities required for autonomous choice.

It is important to note that no data indicates this is occurring. Moreover, available data suggests that the uptake of Wegovy is relatively slow in the pediatric population compared to adults. In the United States, for example, despite an estimated 14.7 million children living with obesity, only 464 pediatric patients across five states—including Michigan, Minnesota, and Wisconsin—were prescribed Wegovy following its approval in January 2022 (Respuat and Terhune 2024). According to a CNBC article, 9 million adults were prescribed Ozempic, Wegovy, and similar obesity and diabetes medications in the last three months of 2022 (Constantino 2023). Uptake rates may change. However, there are several reasons to think that even if they do, this will not make Wegovy the standard first-line treatment for childhood obesity.

First, Wegovy is a costly therapy. In the United States, for example, its listed price is $1,350 for a 28-day supply, amounting to $16,200 per year (Warren 2025). Many countries, including the U.S., do not subsidize it under their national healthcare plans (Singh 2025). Given the expense and the need for sustained treatment, Wegovy is unlikely to be accessible to many families—even as a treatment after other approaches have failed.

Second, prescribing Wegovy to children without first addressing or concurrently addressing healthy lifestyle interventions is at odds with current prescription guidelines. The American Academy of Pediatrics (AAP) recommends that weight loss medications like Wegovy should be part of a comprehensive weight management plan that includes education and lifestyle interventions:

“No current evidence supports weight loss medication use as a monotherapy; thus, pediatricians and other PHCPs [primary healthcare providers] who prescribe weight loss medication to children should provide or refer intensive behavioral interventions for patients and families as an adjunct to medication therapy” (Hampl et al. 2023: 62).

Third, as described above, many parents and children favor pursuing weight loss through traditional lifestyle interventions. This preference is so widespread that Rosenberg refers to it as “common sense.” 8

So, while there is a risk that parents and healthcare providers may prioritize medical intervention over education, potentially compromising children’s autonomy, this does not appear to be a pressing concern. Changes in behavior and attitudes, however, should be monitored—especially if the price of Wegovy decreases and it becomes more accessible.

## The positive case for wegovy: improving children’s autonomy

### Presenting children with more healthcare options

Beyond rejecting the argument from virtue, there are good reasons to think that Wegovy has the potential to support children’s autonomy.

As we described earlier, to exercise autonomy, one must have options and must not be constrained by controlling influences that determine one’s actions. A key point in the argument from virtue speaks to this concern—that parents and caregivers are denying children the option of weight loss through healthy lifestyle interventions by using Wegovy. This appeal to available options, however, also supports prescribing Wegovy. By making Wegovy available, providers reduce prohibitive control and give competent children more options, thereby creating additional opportunities to exercise their autonomy.

Of course, more options are not necessarily beneficial for autonomy or healthcare. An excess of options can overwhelm decision-making and impede autonomy. Including certain options, such as offering medications known to be addictive, may also be harmful and undermine autonomy. The addition of Wegovy, however, represents an important development in pediatric healthcare. As we have argued elsewhere, although several weight-loss alternatives to Wegovy exist—including lifestyle interventions like diet and exercise, other GLP-1 RAs, and various forms of bariatric surgery—none offer a comparable risk profile with similar health and weight loss outcomes (Ryan and Savulescu, manuscript in review).

Daily exercise and nutrition-focused diets typically carry lower risks but are less effective than when combined with Wegovy. As noted earlier, in the STEP TEENS trial, participants receiving Wegovy lost an average of 16.1% of their body weight, compared to 0.6% in the placebo group. Health-risk indicators, including total cholesterol and blood pressure, were also lower with Wegovy than placebo. Other GLP-1 RAs, such as Novo Nordisk’s Saxenda (liraglutide), have similar side effect profiles but are less effective. A recent meta-analysis of three phase 3 trials (296 participants) found that liraglutide did not significantly reduce BMI or body weight compared to placebo in children and adolescents (Cornejo-Estrada et al. 2023). Bariatric surgery—including gastric banding, gastric bypass, sleeve gastrectomy, and duodenal switch—while demonstrating superior weight loss and health improvements, carries a higher risk profile in children due to higher rates of adverse events (Klair et al. 2023).

Given that diet and exercise alone are unlikely to produce the necessary health or weight loss benefits for some children, and that not all children who may benefit from medical intervention require more extreme interventions, such as bariatric surgery, Wegovy may represent an appropriate and effective option for some pediatric patients. As such, Wegovy is an important therapeutic alternative that should be available to children as part of a broader effort to support their individual healthcare choices.^9^

### Modeling autonomous decision-making for children

Some children will not have the capacity to exercise their autonomy by providing informed consent to the use of Wegovy, even if it is made available. This is because decisional competence in healthcare requires a level of cognitive maturity sufficient to understand the available options, anticipate their potential consequences, and evaluate how each aligns with one’s personal goals and values—capacities that may exceed the developmental abilities of many, especially younger children, and this is the case even if they meet the requirements for volitional autonomy.

In the United Kingdom, individuals aged 16 and above are typically presumed competent to make their own healthcare decisions, while those aged 13 and older may be assessed individually for competence (Care Quality Commission 2025). In the United States, standards vary by state, with some jurisdictions recognizing decisional competence starting at ages 12 to 14; even then, youth under 16 are generally encouraged to involve parents in medical decision-making (Sharko et al. 2022).

Given that some children cannot consent to Wegovy as a healthcare option, one might argue that for these individuals, increasing the number of options—specifically the availability of Wegovy—offers limited value for promoting autonomy.

While the availability of more options may not directly enhance younger children’s decisional capacity, offering Wegovy as a healthcare option can nonetheless support and add value to their autonomy. Autonomous action—and the virtues that support it—do not emerge fully formed but develop gradually through experience, reflection, and appropriate guidance. As discussed earlier, these capacities evolve over time, and exposure to meaningful choices within a supportive framework can play a critical role in fostering the skills and dispositions necessary for autonomy.

In healthcare settings, parents have the opportunity to include their children in and model autonomous decision-making. In doing so, both parents and healthcare providers can respond to children’s questions and engage with them in ways that are sensitive to their developmental needs. Through this process, children can gain a deeper understanding of medical concepts and their own healthcare needs, fostering the skills necessary for informed decision-making in the future.

Insofar as it adds another option to consider, including Wegovy in such discussions contributes to a more complex analysis of expected benefits and harms and allows children to engage with more nuanced healthcare choices. It also introduces them to the diversity of medical needs and responses, emphasizing that not all treatments are suitable for all individuals. This recognition of difference is essential to informed decision-making and supports better choices when grounded in meaningful, age-appropriate dialogue.

This is not to suggest that children cannot acquire such knowledge elsewhere, or that Wegovy *must* be available for children to appreciate the diversity of healthcare options. Rather, the point is that Wegovy, when thoughtfully incorporated into pediatric healthcare decision-making and support, can contribute meaningfully to the development of even young children’s autonomy.

### Enhancing impulse control, experience, and bodily autonomy

Another way Wegovy may support children’s autonomy is by enhancing impulse control and motivation in pursuit of their chosen goals. As previously discussed, autonomous action involves acting with intention rather than on impulse. In Harry Frankfurt’s (2018) terms, this entails acting in accordance with one’s “higher-order” volitions—desires about which desires one wants to act upon. One of the mechanisms by which Wegovy operates is by “silencing food noise,” or reducing persistent thoughts and preoccupation with food and hunger. This reduction in intrusive impulses can serve both as an expression of autonomy—by enabling children to act in line with their considered goals—and as a means of fostering autonomy by creating the cognitive space needed for intentional, values-aligned decision-making.

Food noise, particularly in the context of weight loss, is often an unpleasant and intrusive experience. On some accounts, it is not only distracting but can also be deeply distressing, and in certain cases may manifest as a feature of obsessive-compulsive disorder OCD (Sayrany 2024). By helping to silence food noise, Wegovy may support children in achieving their weight loss goals not only by enabling the expression of autonomy and creating cognitive space, but also by making the weight loss process more tolerable—and potentially even enjoyable—by allowing children to engage more freely in activities, including those focused on improving health, without the ongoing struggle of food-related rumination. For these reasons, we have argued elsewhere that Wegovy should be understood as a form of motivational enhancer ( Ryan and Savulescu 2025).

### Maintaining open futures

One final way that Wegovy may support children’s autonomy is by keeping their futures open. Recall that, on Feinberg’s view, children have a moral right to an “open future”—that is, broadly speaking, a right to reach maturity with “as many open options, opportunities, and advantages as possible.” Feinberg’s view has been criticized on the grounds that it suggests autonomy depends on the number of options available, whereas neither morally abhorrent nor trivially different options enhance autonomy (Millum 2014).

Happily, there are at least three ways in which Wegovy may help preserve open futures that are morally acceptable and more than trivially different from other available options. The first is by reducing the risk of developing obesity-related health conditions, such as heart disease and non-alcoholic fatty liver disease, later in life. By lowering the likelihood of such illnesses, Wegovy improves children’s chances of living with more opportunities, including those not constrained by chronic health issues.

The second is by providing motivational support. By reducing food noise and freeing up mental space, Wegovy enables children to pursue other goals that may build a foundation for future opportunities. Children, for example, may be better able to concentrate at school or participate in leisure activities that promote physical and mental health—activities valuable not only in childhood but also for the opportunities they may create in adulthood.

The third way Wegovy may support open futures—if administered responsibly and with appropriate support for children’s education and decision-making capacities—is by engaging children in decision-making processes that foster their autonomy. By modeling sound decision-making and teaching healthy lifestyle practices—either as a precursor to or alongside Wegovy—children may develop the capacity to exercise autonomy, particularly in healthcare, in ways that help sustain an open future.

## Barriers to supporting children’s autonomy & wegovy

We have argued so far that the argument from virtue fails because weight loss with Wegovy is not an easy way out, and because neither weight loss itself nor effort is an inherently valuable endeavor. We have also argued that Wegovy has the potential to support children’s autonomy in several important ways. These include keeping children’s future options open; providing opportunities for parents and healthcare providers to model autonomous decision-making and offer education that promotes healthy lifestyles and successful agency; expanding the range of options available to children, thereby increasing the ways they can exercise their autonomy; and enhancing children’s impulse control and motivation toward their chosen ends (Tables 1, 2, 3, 4).

**Table 1:** Wegovy: summary of health benefits and risks in pediatric use

Category	Details
Approved Use	Obesity in children aged 12+, or overweight with comorbidities
Dosage	Weekly injection: starts at 0.25 mg, increases to 2.4 mg maintenance dose
Mechanism of Action	Suppresses appetite via gastric emptying delay and brain signalling related to hunger and satiety (“food noise”)
Reported Benefits	− 16.1% average weight loss in 68 weeks (vs. 0.6% placebo) − 44% reclassified to normal/overweight BMI (vs. 12.1%). Improved cardiovascular and NAFLD markers, etc.
Mild to Moderate Side Effects	Nausea, vomiting, diarrhea, constipation, bloating, abdominal pain, headache, loss of lean muscle mass, exercise-related weakness
Mild/Mod. Side Effect Incidence	62% of Wegovy group vs. 42% placebo
Serious Side Effects (Rare)	Gastroparesis, anhedonia, pancreatitis, thyroid C-cell tumors, kidney failure, gallbladder disease, diabetic retinopathy
Trial Discontinuation	5% of semaglutide group vs. 4% placebo due to adverse events
Post-Treatment Weight Regain	~Two-thirds of weight regained within 1 year after stopping treatment
Long-Term Risks in Youth	Still unclear; same risks as adults listed in product warnings. Malnutrition also noted as a concern

**Table 2:** Comparison of pediatric weight loss interventions

Intervention	Effectiveness	Risk Profile	Notes
Wegovy (semaglutide)	Average 16.1% weight loss in STEP TEENS trial; improved cholesterol and blood pressure	Mild to moderate side effects common (e.g. nausea); serious side effects rare	Most effective among non-surgical options; requires monitoring and support
Lifestyle interventions (diet + exercise)	Minimal average weight loss (0.6% in STEP TEENS trial control group); more effective when combined with Wegovy	Very low risk	Autonomy-promoting but insufficient alone for some children with obesity
Saxenda (liraglutide)	Not significantly effective in reducing BMI or body weight in youth	Similar side effects to Wegovy	Less effective than Wegovy despite similar mechanism
Bariatric surgery	Highly effective; significant and sustained weight loss; improved health outcomes	High risk profile in children: more adverse events	Considered a last resort due to surgical risk and invasiveness

**Table 3:** Wegovy and children’s autonomy: benefits and barriers

Potential Autonomy-Supporting Benefits	Barriers to Realization
Keeps future options open by reducing long-term health limitations related to obesity.	**Structural inequality**: Limited access to healthcare, education, and leisure time constrains real choice.
Models autonomous decision-making when offered with age-appropriate explanations and support.	**Framing as an “easy way out”** may reinforce moral judgment and diminish perceived agency.
Expands available options, allowing children to choose treatments that align with their needs/goals.	**Weight stigma** may pressure children to take or reject Wegovy, undermining true voluntary choice.
Supports impulse control and motivation, enabling children to act more intentionally toward their ends.	**Moralized discourse** around Wegovy (e.g. “miracle drug”) may coerce both use and non-use.
Facilitates autonomy development through informed, supported healthcare engagement.	**Shame as a coercive force** can compromise autonomy from both directions: use or non-use invites judgment.
	**Resource constraints**: Time, clinician training, and medication access may be lacking in overstretched systems.

**Table 4:** Wegovy and children’s autonomy: summary of recommendations

Recommendation	Rationale	Conditions/Notes
Further research on Wegovy’s long-term effects in children	To support informed decision-making and safeguard pediatric health	Research should address both physical health outcomes in pediatric community and impacts on autonomy and agency
Invest in structural reforms to address obesity’s root causes	To reduce reliance on medicalized interventions and support genuine choice	Reforms should target stigma, poverty, and health system resource limitations
Prioritize lifestyle interventions before prescribing Wegovy	To promote autonomy by enabling children to achieve health goals without medicalization	Prescribe Wegovy only if lifestyle approaches have failed or are unsuitable for the child
Prescribe Wegovy before considering invasive treatments	To minimize risks of physical harm and protect future opportunities	Wegovy should precede more invasive options, such as bariatric surgery
Prescribe Wegovy in ways that respect children’s autonomy	To align with the ethical priority of promoting autonomy	Requires age-appropriate information, decisional support, and a supportive environment
Allow treatment when benefits to health and autonomy are clear, even under non-ideal conditions	To recognize practical realities while aiming to support the child’s well-being and agency	Requires individualized benefit–harm analysis, ongoing monitoring, and support

While these benefits are promising, it is important to note several potential barriers to their realization. As we have explained elsewhere, some commentators have expressed concern that Wegovy may be embraced not only by individuals as an “easy way out,” but also by governments as a convenient alternative to addressing the underlying structural causes of obesity ( Ryan and Savulescu 2025). These include resource inequalities in healthcare, education, and leisure time—resources that enable individuals to exercise their autonomy by making informed, supported healthcare and lifestyle decisions. Moreover, if governments do not address these inequalities, the availability of Wegovy to well-resourced families could exacerbate health care inequalities.

Other commentators have also rightly worried that if weight stigma is not addressed, the widespread use of Wegovy may exacerbate it, leading to further harm and coercion. As Shanouda and Orsini (2023) describe, current discourse around Ozempic frames obesity as “a vice of the lazy” that must be “stamped out” and “cured.” With Wegovy on the market, they argue, this discourse has shifted fat-shaming to an unprecedented level. It now dominates media and interpersonal narratives through moralized discussions of Wegovy as a “miracle drug” that will “end obesity.”

The concern for autonomy here is that this discourse may coerce adults and children into taking Ozempic or Wegovy and further shame those who do not. Importantly, if Rosenberg is correct, given that many people appear to endorse the “common sense” argument from virtue, those who take Wegovy (or are perceived to take it) will also be subject to shame for “taking the easy way out.” Insofar as shame operates as a coercive force and weight stigma remains unaddressed, autonomy is compromised from both directions—to use a colloquial expression, you are damned if you do, and damned if you don’t.

A further concern is limited resources. It is our view that, to protect and promote children’s autonomy in healthcare, Wegovy should be presented to a child and their family as a treatment option when the child stands to benefit in terms of both health and autonomy. It should also be offered and prescribed in ways that support autonomy—through age-appropriate explanations and decisional scaffolding where necessary. Prescribing the medication, along with providing the necessary support, requires resources such as time, training—particularly for clinicians not already experienced in supporting children’s autonomy—and access to the medication itself. For healthcare systems already struggling to meet existing demands, fulfilling these requirements may place an additional, potentially prohibitive strain. This strain, moreover, may be further exacerbated by the potential for abuse that Wegovy presents. As Cooper et al. (2023) explain, the fragility of adolescent self-esteem, the pervasive marketing of Wegovy on social media, and the high prevalence of eating disorders among U.S. youth make the introduction of medications like Wegovy a “perfect storm” for abuse. This is particularly worrisome because of its potential to contribute to or worsen eating disorders such as anorexia nervosa—conditions that can be life-long and significantly curtail a person’s autonomy in ways similar to addiction and its treatment. 10 To protect against this, patients would require evaluation for risk of abuse, as well as greater supervision and potentially intervention.^11^

## Supporting children’s autonomy with medicalized weight loss: recommendations

How then should we proceed with prescribing Wegovy to children?

If respecting and promoting children’s autonomy is a priority, there are ways to do so that are compatible with prescribing Wegovy. Important social, structural, and resource barriers must still be addressed to ensure children can make uncompromised healthcare decisions as described above. Knowledge gaps regarding the long-term effects of Wegovy, especially on developing bodies, must also be addressed.

These considerations highlight the need for further research into the effects of Wegovy on pediatric populations to support informed decision-making and improve pediatric health. Additionally, investment in structural reform is required to address the root causes of obesity-related health issues, stigmatization, and healthcare resource limitations. Given the potential agentic and health-related benefits Wegovy may offer children, it is our view that—even under non-ideal conditions where research and social change are lacking—treatment may still be appropriate when a child stands to benefit in terms of both health and autonomy. Given the unknowns, any determination of benefit should be reached only after a careful analysis of the expected benefits and harms that considers the individual needs and goals of the child, with ongoing monitoring and support.

It is also our view that Wegovy should be offered as a healthcare option only after traditional lifestyle interventions to promote health have failed or when such interventions are deemed unsuitable for the child—for example, because they would be dangerous or unsuitable given the child’s needs. Priority should be given to lifestyle interventions, however, not because healthcare through these means is inherently more virtuous, but because, if successful, they allow children to meet their healthcare goals without medicalized intervention and dependence—a freedom that promotes autonomy.

Wegovy should also be prescribed before more invasive procedures, such as bariatric surgery, are pursued to reduce the risks of physical and agentic harm and to safeguard the child’s future opportunities.

Moreover, given the centrality of respect for autonomy in medical ethics, if Wegovy is presented and prescribed to children and families, this should be done only in ways that respect their autonomy—with age-appropriate explanations, decisional scaffolding where necessary, and within a supportive environment. If such conditions cannot be met, it would need to serve some higher-order value that outweighs respect for patient autonomy, which should be a very high standard to satisfy.

We have focused here on some of the key ethical issues related to Wegovy and children’s autonomy. There are, however, other dimensions to consider. One important dimension pertains to Wegovy’s ability—like that of other GLP-1 RAs—to control a child’s impulses, particularly in cases where the child is not yet capable of consenting to medical intervention. With such medications, not only is another party determining a child’s healthcare treatment, but they are also, in some sense, influencing that child’s very *will*. While this may offer motivational benefits, it also raises important questions about the limits of morally acceptable healthcare interventions and the legitimate boundaries of parental authority over the development and expression of children’s autonomy. Our preliminary view is that GLP-1 RAs appear no more ethically problematic than widely accepted pediatric interventions, such as methylphenidate for treating Attention-Deficit/Hyperactivity Disorder (ADHD). However, a full discussion of these concerns is warranted and will be the subject of future work.

## Data Availability

Not applicable.

## Footnotes

1 Thank you to Prof. Colin Klein for insight on this point.

2 For an extended discussion of the health benefits see Ryan and Savulescu 2025).

3 For a summary of the health benefits and risks of Wegovy in pediatric populations, see Table 1. 

4 The kind of autonomy that Mullin refers to is “local autonomy” rather than “global autonomy.” Local autonomy is “autonomy in action, in the particular choices and actions that one makes from day to day.” In contrast, global autonomy—the kind of autonomy expressed in maturity—is “a trait characterizing a substantial period of one’s life and all significant domains within it,” reflecting one’s established identity (Mullin 2019: 231). It is Mullin’s view that while young children typically do not possess global autonomy until reaching maturity, they can nevertheless exercise local autonomy.

5 Other philosophers who have in recent years recognized that young children may be capable of autonomy include Sarah Hannan (2019), Paul Bou-Habib and Serena Olsaretti (2014), and Betzler (2015).

6 For an extended discussion on self-respect in childhood, see Ryan (2023).

7 For more on weight and class stigma see Tomiyama et al. (2018), Mensinger et al. (2018), and Oswald (2024), and Shanouda and Orisini (2023).

8 Importantly, not all parents and children in the study by van Geelen et al. (2013) favored lifestyle interventions. Van Geelen et al. found that perceptions of a need to lose weight the “right way” corresponded to a belief that individuals could influence their weight—through a changed mindset or willpower, for example. Those more willing to engage in medical interventions, van Geelen et al. (2013: 227, 235) claim, showed little awareness of their role in the success or failure of that intervention. Interestingly, the disparity in beliefs had implications not only for willingness to engage in the intervention but also for the ability to give informed consent and, in turn, uptake rates. Those unwilling to engage because they believed it was the “wrong way” to lose weight did not increase uptake, and those who were willing but did not understand their role also failed to meet conditions for informed consent. It is unclear what further motivations parents or children had for preferring lifestyle interventions when they did, but a concern with long-term harms, for example, is compatible with their decision making.

9 For a comparative summary of pediatric weight loss interventions, see Table 2. 

10 On impact on autonomy in treatment see Healy (2025), and in condition see Barbarich-Marsteller et al. (2011).

11 For a summary of the benefits and barriers to Wegovy supporting children’s autonomy, see Table 3. 
